# Synthesis and Characterization of Anti-HER2 Antibody Conjugated CdSe/CdZnS Quantum Dots for Fluorescence Imaging of Breast Cancer Cells

**DOI:** 10.3390/s91109332

**Published:** 2009-11-19

**Authors:** Dhermendra K. Tiwari, Shin-Ichi Tanaka, Yasushi Inouye, Keiko Yoshizawa, Tomonobu M. Watanabe, Takashi Jin

**Affiliations:** 1 WPI Immunology Frontier Research Center, Osaka University, Yamada-oka 1-3, Suita, Osaka 565-0871, Japan; E-Mails: mdkdragon@gmail.com (D.T.); kyoshizawa@fbs.osaka-u.ac.jp (K.Y.); twatanabemeister@gmail.com (T.W.); 2 Graduate School of Frontier Biosciences, Osaka University, Yamada-oka 2-1, Suita, Osaka 565-0871, Japan; E-Mails: s-tanaka@ap.eng.osaka-u.ac.jp (S.T.); ya-inoue@ap.eng.osaka-u.ac.jp (Y.I.)

**Keywords:** quantum dots, HER2, antibody, cellular imaging, breast cancer cell

## Abstract

The early detection of HER2 (human epidermal growth factor receptor 2) status in breast cancer patients is very important for the effective implementation of anti-HER2 antibody therapy. Recently, HER2 detections using antibody conjugated quantum dots (QDs) have attracted much attention. QDs are a new class of fluorescent materials that have superior properties such as high brightness, high resistance to photo-bleaching, and multi-colored emission by a single-light source excitation. In this study, we synthesized three types of anti-HER2 antibody conjugated QDs (HER2Ab-QDs) using different coupling agents (EDC/sulfo-NHS, iminothiolane/sulfo-SMCC, and sulfo-SMCC). As water-soluble QDs for the conjugation of antibody, we used glutathione coated CdSe/CdZnS QDs (GSH-QDs) with fluorescence quantum yields of 0.23∼0.39 in aqueous solution. Dispersibility, hydrodynamic size, and apparent molecular weights of the GSH-QDs and HER2Ab-QDs were characterized by using dynamic light scattering, fluorescence correlation spectroscopy, atomic force microscope, and size-exclusion HPLC. Fluorescence imaging of HER2 overexpressing cells (KPL-4 human breast cancer cell line) was performed by using HER2Ab-QDs as fluorescent probes. We found that the HER2Ab-QD prepared by using SMCC coupling with partially reduced antibody is a most effective probe for the detection of HER2 expression in KPL-4 cells. We have also studied the size dependency of HER2Ab-QDs (with green, orange, and red emission) on the fluorescence image of KPL-4 cells.

## 1. Introduction

Tumor cells show overexpression of HER2 (human epidermal growth factor receptor 2) in approximately 20%−30% of breast cancer patients [1,2]. Thus, molecular-targeted therapy for HER2 has a tremendous impact on breast cancer treatment. Anti-HER2 antibodies inhibit the growth of HER2-overexpressing breast cancer cells, and the anti-HER2 antibody therapy is most effective in HER2-positive breast cancer patients [3-5]. The early detection of HER2 status in breast cancer patients is crucial for the effective implementation of anti-HER2 antibody therapy. Currently, immunohistochemistry (IHC) and fluorescence *in situ* hybridization (FISH) are the most widely used methods for determining HER2 status in breast cancer patients [6]. IHC is a semi quantitative technique and its accuracy depends on the proficiency of the pathologist [7,8]. Although FISH analysis is more sensitive and quantitative than IHC in determining HER2 status, this method is complicated, expensive and time-consuming [9,10].

In a few years, HER2 detection using quantum dot (QD) based fluorescent probes have attracted much attention [11-22]. Compared to traditional fluorescent organic dyes and proteins, QDs have superior properties such as high brightness, high resistance to photo-bleaching, and multi-colored emission by a single source excitation. Several groups have reported staining of HER2 overexpressing breast cancer cells using anti-HER2 antibody conjugated QDs (HER2Ab-QDs) [11-18,20-22]. Liu *et al.* first showed the utility of QDs for immunofluorescent staining of HER2 in breast cancer SK-BR-3 cells, and they demonstrated that the QD-based fluorescent probes offer substantial advantages over organic dyes in multiplex target detection [11]. Recently, Li *et al.* have developed QD-based immunofluorescence technology for the quantitative determination of HER2 expression in breast cancer tissues [19].

Several kinds of conjugation methods can be used for the development of HER2Ab-Qds [23]. The most popular conjugation method involves the use of a zero-length crosslinker, EDC (1-ethyl-3-(3-dimethyl-aminopropyl)carbodiimide hydrochloride for the formation of amide bonds between carboxyl groups (introduced to the surface of QD) and primary amines of antibody [23]. In EDC coupling method, there is a possibility that the antigen binding sites of the antibodies are blocked by the non-selective formation of amide bonds at the vicinity of F_ab_ region of antibody [16,23]. To avoid this, SMCC (succinimidyl 4-(*N*-maleimidomethyl)cyclohexane-1-carboxylate) coupling reaction can be used as a substitute for the selective conjugation of partially reduced antibodies to QDs. In this case, sulfhydyl groups (at F_c_ region) of reduced antibodies bind to the maleimide groups of the SMCC coupled QDs [23]. Besides these two methods, noncovalent conjugation of streptavidin coated QDs and biotinylated antibodies can be used for preparing HER2Ab-QDs [16,22]. However, the relationship between antibody conjugation methods and effectiveness of the resulting HER2Ab-QDs for cellular imaging is not clear.

The objective of this work was to synthesis anti-HER2 antibody conjugated QDs (HER2Ab-QDs) prepared by different coupling methods [23] and to examine their staining abilities for HER2 overexpressing breast cancer cells. We synthesized three types of HER2Ab-QDs using the EDC/sulfo-NHS (3-sulfo-*N*-hydroxysuccinimide sodium salt), iminothiolane/sulfo-SMCC, and sulfo-SMCC coupling methods. As water-soluble QDs for the conjugation of anti-HER2 antibody, we used glutathione coated CdSe/CdZnS QDs (GSH-QDs) [24] with fluorescence quantum yields of 0.23∼0.39 in aqueous solution. In the first type of HER2Ab-QDs, primary amines of anti-HER2 antibody are non-selectively conjugated to the carboxy groups of GSH-QDs via EDC/sulfo-NHS coupling. In the second type of QDs, iminothiolane modified anti-HER2 antibodies non-selectively conjugated to the surface of GSH-QDs with maleimide groups of SMCC coupled GSH-QDs. In the third type, SMCC coupled GSH-QDs selectively conjugated to the sulfhydryl groups of reduced antibodies. The size, surface structure, and apparent molecular weights of GSH-QDs and HER2Ab-QDs were characterized by using dynamic light scattering (DLS), fluorescence correlation spectroscopy (FCS), atomic force microscopy (AFM), and size-exclusion HPLC. Staining ability of the HER2Ab-QDs for HER2 overexpressing breast cancer cells (KPL4-cells) was examined by laser confocal fluorescence imaging. We found that the HER2Ab-QDs prepared by SMCC coupling with reduced antibodies were most effective in concern to the stability of the antibody conjugated complex and the detection of HER2 expression in breast cancer cells. We have also examined the size dependency of HER2Ab-QDs (with green, orange, and red emission) on the cellular uptake and localization.

## 2. Results and Discussion

### 2.1. Surface Modification of QDs with Glutathione (GSH)

The synthesis of highly fluorescent QDs is generally performed in organic solvents such as trioctylphosphine oxide (TOPO) and hexadecylamine (HDA) at high temperature (>200 °C) [25-29]. The resulting QDs are coated with hydrophobic organic compounds so that they are not soluble in water. For bioconjugation, QD surface should be modified with functional groups such as carboxyl groups and amines. In this study, we used glutathione (GSH, reduced form) as a coating agent for the surface modification of TOPO/HDA capped QDs [30-32]. GSH is a natural thiol compound (γ-*L*-glutanyl-*L*-cystenylglycine) that exists in most organs at mM levels [33,34], and the compound is not harmful like synthetic thiol compounds (e.g. mercaptoacetic acid and mercapotethanol). In addition, it has been shown that the GSH has a property to detoxify Cd^2+^ ions in cellular level due to its chelating capability [35].

**Figure 1.**
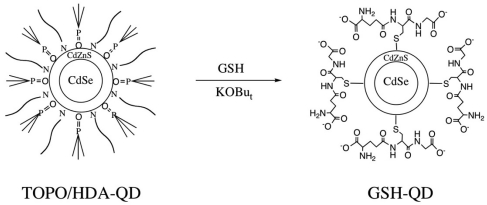
Schematic representation for the surface coating procedure with GSH. GSH coating is performed in a mixture of THF-water at 60 °C. Potassium *t*-butoxide (KOBu_t_) is used for deprotonation of the carboxyl groups of GSH.

Figure 1 shows the surface coating procedure for preparing GSH coated QDs (GSH-QDs). First, TOPO/HDA coated QDs (TOPO/HDA-QDs) are dispersed to tetrahydrofuran (THF). Then the aqueous solution of GSH is added to the THF solution of QDs for ligand-exchange with GSH. As a result of ligand exchange, GSH-coated QDs precipitate in THF-water solution. After deprotonation of the carboxyl groups at the QD surface, GSH-coated QDs are easily dissolved in water. Since the GSH-coated QDs have both carboxyl groups and primary amines at their surface, various coupling agents can be used for antibody conjugation. In this study, we synthesized three types of antibody-conjugated GSH-QDs by using EDC/sulfo-NHS, iminothiolane/SMCC, and SMCC coupling agents.

### 2.2. Characterization of GSH-QDs

#### 2.2.1. Fluorescence quantum yields

**Figure 2.**
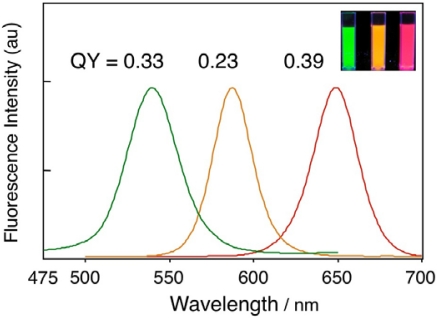
Fluorescence spectra and quantum yields of GSH-QDs that have emission peaks at 540 nm (green), 585 nm (orange), and 650 nm (red) in 10 mM PBS buffer. Inset shows a fluorescence image of three types of GSH-QDs in PBS buffer under irradiation of a UV light (365 nm).

In cellular imaging, the brightness of fluorescent probes is crucial for obtaining clear images with a high signal to noise ratio, because cells contain intrinsic fluorophores such as aromatic amino acids, neurotransmitters and porphyrins that emit in the visible region. Figure 2 shows the fluorescence spectra of GSH-QDs with the values of fluorescence quantum yields in 10 mM PBS. The fluorescence emission efficiencies of TOPO/HDA capped CdSe/CdZnSe QDs were very high and their quantum yields were 0.60, 0.45 and 0.72 in chloroform for green-, orange-, and red- emitting QDs, respectively. After the ligand-exchange with GSH, the quantum yields decreased to be 0.33, 0.23 and 0.39 for green-, orange-, and red- emitting GSH-QDs, respectively. GSH-coating retained *ca.* 50% of fluorescence efficiency after the surface modification. When the surface of the QDs was modified with MAA and MPA, the quantum yields of the QDs were less than 0.1. The surface modification of TOPO/HDA capped QDs with GSH is very simple and useful for preparing highly fluorescent water-soluble QDs.

#### 2.2.2. Hydrodynamic size and dispersibility of QDs

**Figure 3.**
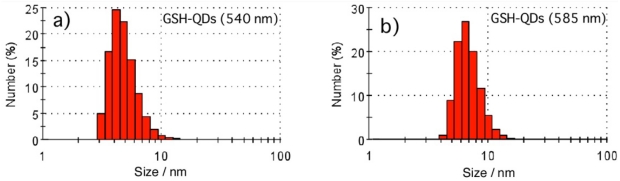
Dynamic light scattering histogram for GSH-QDs in PBS buffer: (a) GSH-QDs (540 nm) and (b) GSH-QDs (585 nm).

Hydrodynamic size and dispersibility of QDs are very important properties for their applications to cellular imaging. The particle size of QDs affects endocytosis or limits access to receptors of interest on cellular membranes [24,36,37]. In living tissues, QD particle size affects biodistribution and pharmacokinetics [38,39]. Hydrodynamic size of GSH-QDs was evaluated by using dynamic light scattering (DLS) and fluorescence correlation spectroscopy (FCS). Figure 3 shows DLS histogram for green- and orange-emitting QDs in PBS buffer. The histogram shows that the GSH-QDs are monodisperse particles, and their hydrodynamic diameters are 4.5 ± 0.6 nm and 5.9 ± 0.5 nm for green- and orange-emitting QDs, respectively. For the red-emitting GSH-QDs with a 650 nm emission peak, reliable DLS data was not obtained due to the overlapping between QD fluorescence around 650 nm and the light scattering from a 633 nm He/Ne laser. Hence, the hydrodynamic size of the red-emitting GSH-QDs (650 nm) was determined by using FCS [40-44].

**Figure 4.**
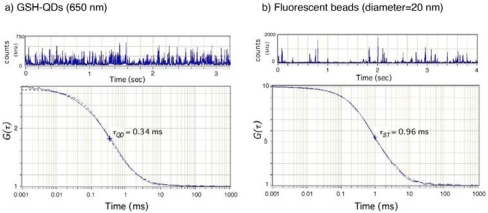
Fluorescence autocorrelation curves *G(τ)* for red-emitting GSH-QDs (650 nm) and standard fluorescent beads (20 nm in diameter) in 10 mM PBS. The autocorrelation curves are fitted by using a single-component diffusion model (broken lines).

Figure 4 shows the fluorescence autocorrelation curves *G(τ)* for GSH-QDs (650 nm) and fluorescent latex beads (20 nm in diameter) in 10 mM PBS. Both the *G(τ)* curves can be fitted according to a single-component diffusion model [equation (2) in the Experimental section], indicating that GSH-QDs and fluorescent latex beads are monodisperse in the PBS buffer. From the fitted curves based on a single-component diffusion model, the diffusion times (τ) are determined to be 0.33 ms and 0.96 ms for GSH-QDs (650 nm) and fluorescent latex beads (20 nm in diameter), respectively. Using the values of τ, the hydrodynamic diameter of the GSH-QDs (650 nm) is calculated to be 6.9 ± 0.5 nm. The hydrodynamic size of commercially available QDs (Invitrogen, Qdot 655 ITK) with a 655 nm peak emission is reported as *ca.* 19 nm in diameter [45]. GSH-QDs (650 nm) prepared by our method is at least two times smaller compared to the commercial QDs.

#### 2.2.3. Apparent molecular weights of GSH-QDs

**Figure 5.**
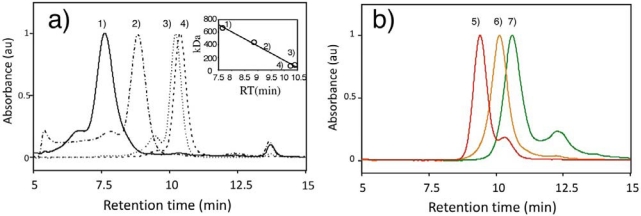
Size-exclusion HPLC chromatograph for standard proteins (a) and GSH-QDs using a TSKgel G4000SW column with 10 mM PBS buffer as an eluent; 1) thyroglobulin (670 kDa), 2) ferritin (450 kDa), 3) bovine serum albumin (66 kDa), 4) transferrin (80 kDa), 5) GSH-QD (650 nm), 6) GSH-QD (585 nm), and 7) GSH-QD (540 nm).

Furthermore, we evaluated the apparent molecular weights of GSH-QDs using size-exclusion HPLC. Figure 5 shows a size-exclusion HPLC chromatograph for standard proteins (thyroglobulin: 670 kDa, ferritin: 450 kDa, transferrin: 80 kDa and bovine serum albumin: 66 kDa) and GSH-QDs. The relationship between molecular weights of the standard proteins and their retention times is almost linear as shown in the inset of Figure 5a. From this relationship, apparent molecular weights of GSH-QDs can be determined. The result is summarized in Table 1 together with the values of quantum yield and hydrodynamic size. The apparent molecular weight of GSH-QDs increases with increasing their hydrodynamic diameter: 75 kDa, 150 kDa, and 300 kDa for GSH-QD (540 nm), GSH-QD (585 nm), and GSH-QD (650 nm), respectively The molecular weight of GSH-QD (585 nm) is *ca.* three times larger than that of a green fluorescent protein, GFP (27 kDa), while the size of QD is comparable to that of GFP having a cylindrical shape with 4.2 nm long and 2.4 nm in diameter [46]. The molecular weight of GSH-QD (585 nm) is comparable to that of antibody immunoglobulin G, IgG (160 kDa). In the case of GSH-QD (650nm), its molecular weight is two-times larger than that of IgG, while the size of QD is comparable to that of IgG (14.5 × 8 × 4 nm^3^) [47,48].

#### 2.2.4. Stability of GSH-QDs in aqueous solution

Colloidal stability of QDs around the neutral pH is necessary for biomolecule conjugation, because most of the conjugation reactions are performed in physiological buffers. When an aqueous solution of GSH-QDs (PBS buffer, pH = 7.2) was preserved in the dark at 4 °C, monodispersibility of the QDs was maintained at least for two months. The monodispersibility of the QDs was analyzed by using DLS and FCS (data not shown). In contrast, when the aqueous solution of GSH-QDs was preserved under room light at room temperature, the QDs aggregated within one month. This instability of the QDs can be explained by photooxidation of GSH, which may induce the degradation of the surface of GSH-QD to aggregate QDs [49].

### 2.3. Conjugation of Anti-HER2 Antibody to GSH-QDs

**Figure 6.**
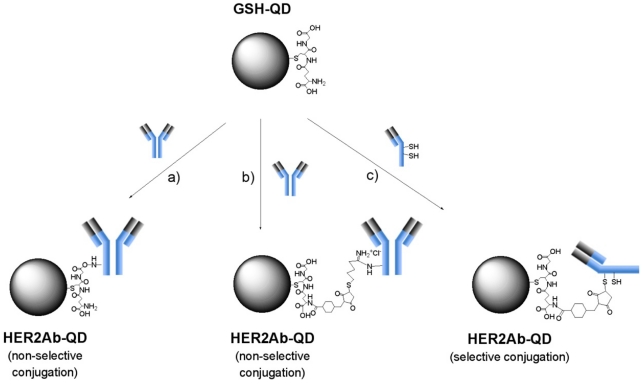
Schematic representation for the coupling reactions between GSH-QDs and anti-HER2 antibodies: a) EDC/sulfo-NHS, b) iminothiolane/sulfo-SMCC, and c) sulfo-SMCC coupling.

There are various methods for the conjugation of antibodies to functionalized QDs with carboxyl groups and/or primary amines [16,23,51]. We prepared three types of anti-HER2 antibody conjugated GSH-QDs (HER2Ab-QDs) as shown in Figure 6. In all of the HER2Ab-QDs, antibodies are covalently bound to the surface of QDs. In the case of EDC/sulfo-NHS coupling, NHS groups are introduced to the surface of GSH-QDs and the NHS groups react non-selectively with the primary amines of antibody to form amide bonds. For the iminothiolane/SMCC coupling, the primary amines of antibody are non-selectively modified with iminothiolane to introduce sulfhydryl groups to the antibody. The sulfhydryl groups of antibody react with the maleimide groups at the surface of SMCC activated QDs. In this coupling method, antibody orientation at the surface of QDs cannot be fixed due to the non-selective conjugation of antibodies to QDs. In the case of SMCC coupling with reduced antibodies (prepared by DTT or cysteamine), the antigen binding sites of antibodies face outward, because the sulfhydryl groups of reduced antibodies bind to the maleimide groups at the surface of SMCC coupled QDs.

**Figure 7.**
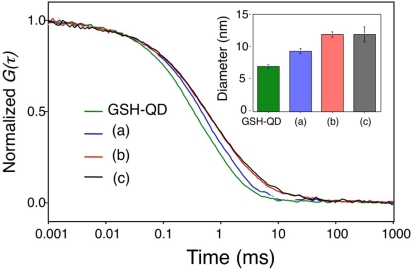
Fluorescence autocorrelation curves for red-emitting GSH-QDs and HER2Ab-QDs prepared by different coupling methods: (a) HER2Ab-QD (sulfo-SMCC), (b) HER2Ab-QD (EDC/sulfo-NHS), and (c) HER2Ab-QD (iminothiolane/sulfo-SMCC). Inset shows hydrodynamic diameters of the QDs in 10 mM PBS buffer.

The binding of antibody molecules to GSH-QDs was confirmed by the measurements of the hydrodynamic size and shape of QDs by using FCS and AFM. Figure 7 shows fluorescence autocorrelation curves of red-emitting GSH-QDs and HER2Ab-QDs prepared by three different coupling methods. The diffusion times of all types of HER2Ab-QDs are larger than that of GSH-QDs, showing that the antibodies are bound to GSH-QDs. From the values of diffusion times, hydrodynamic diameters are determined to be 7.0 ± 0.3 nm for GSH-QDs, 9.4 ± 0.5 nm for HER2Ab-QDs (SMCC coupling), 12 ± 0.4 nm for HER2Ab-QDs (EDC/sulfo-NHS coupling), and 12 ± 1.2 nm for HER2Ab-QDs (iminothiolane/SMCC coupling). These results support that the whole bodies of antibody are bound to GSH-QDs in case of HER2Ab-QDs (EDC/sulfo-NHS coupling) and HER2Ab-QDs (iminothiolane/SMCC coupling), while only half bodies of antibody are bound to GSH-QDs in the case of HER2Ab-QDs (SMCC coupling).

**Figure 8.**
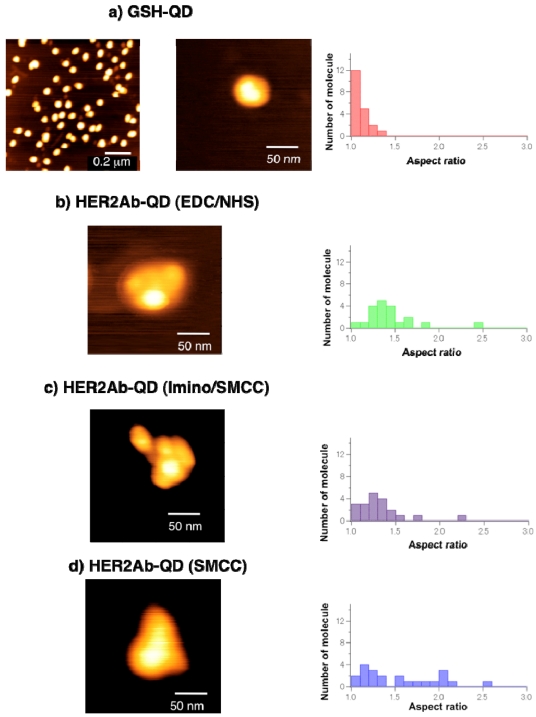
AFM images (left panel) and the histogram of aspect ratio (right panel) of red-emitting GSH-QDs a) and HER2Ab-QDs prepared by the coupling reaction using b) EDC/sulfo-NHS, c) iminothiolane/sulfo-SMCC, and d) sulfo-SMCC.

AFM images also confirmed the binding of antibodies to GSH-QDs (Figure 8). The images of GSH-QDs show spherical shapes, while all three types of HER2Ab-QDs show non-spherical shapes or somehow elongated shapes. Then, we compared GSH-QD structure coupled with different type of antibodies by using the aspect ratio. The aspect ratio of GSH-QDs was almost 1.0 [Figure 8(a)]. The AFM images of HER2Ab-QDs indicate that a few molecules of antibodies are bound to the surface of GSH-QDs and their aspect ratios were also changed. [Figure 8b,c] The aspect ratios of HER2Ab-QDs, which were prepared by EDC/NHS and iminothiolane/SMCC coupling methods, were 1.3 ± 0.2 and almost same. Since the size of anti-HER2 antibody (IgG) is comparable to the size of GSH-QDs (650 nm), steric hindrance between antibody molecules bound at the QD surface may limit the number of antibodies that bind to the surface of QD. The comparison among the shapes of all three types of HER2Ab-QDs indicates that the whole bodies of antibody are bound to the QD surface in HER2Ab-QDs (EDC/NHS) and HER2Ab-QDs (iminothiolane/SMCC). In contrast, only half bodies of antibody are bound to the QD surface in case of HER2Ab-QDs prepared by SMCC coupling to reduced antibodies. As the steric hindrance of half bodies of antibody was less than that of whole bodies of antibody, the prepared HER2Ab-QDs had the various aspect ratio [Figure 8(d)]. The changing of aspect ratio indicated that the binding of antibodies to GSH-QDs.

### 2.4. Confocal Fluorescence Imagimg of KPL-4 Breast Cancer Cells

**Figure 9.**
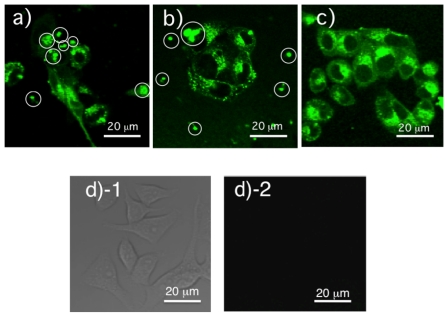
Fluorescence images of KPL-4 cells in the presence (a-c) and absence (d) of green-emitting (540 nm) HER2Ab-QDs prepared by using three different coupling agents: a) EDC/sulfo-NHS, b) iminothiolane/sulfo-SMCC, and c) sulfo-SMCC. PBS solutions (20 nM) of the HER2Ab-QDs were incubated with KLP-4 cells for 30 min at 37 °C. QDs were excited at 405 nm and confocal images were taken with a 490-540 nm filter. Green spots in white circles are attributed to QD aggregates. KPL-4 cells in the absence of green-emitting QDs show no cellular autofluorescence signals: differential interference contrast image (d-1) and fluorescence image (d-2).

We first examined staining abilities and colloidal stabilities of three types of HER2Ab-QDs for fluorescence imaging of KPL-4 human breast cancer cells. Figure 9 shows confocal fluorescence images of KPL-4 cells after 30 min incubation of HER2Ab-QDs (green, 540 nm) prepared by three different coupling methods. All three types of HER2Ab-QDs clearly stained the KPL-4 cells. However, in the case of the HER2Ab-QDs prepared by using EDC/sulfo-NHC and iminothiolane/sulfo-SMCC coupling, QD aggregations were observed in the images (Figure 9a,b). HER2Ab-QDs prepared by SMCC coupling to reduced antibodies showed no aggregation of the QDs and the cells were intensely stained compared to other two types of HER2Ab-QDs (Figure 9c). QD aggregations observed in Figure 9a,b may have resulted from the instability of HER2Ab-QDs in the culturing medium (DMEM). The fluorescence image of KPL-4 cells (Figure 9d-2) in the absence of QDs shows no fluorescence signals, indicating that the fluorescence signals in Figure 9a-c dose not result from autofluorescence of KPL-4 cells. Among all three types of QDs, HER2Ab-QDs conjugated by SMCC coupling method was most stable and effective as a fluorescent probe for imaging of KPL-4 cells. It should be noted that the green-emitting HER2Ab-QDs are distributed in cytosol as well as cell membrane, indicating that the cellular uptake of HER2Ab-QDs occurs by macropinocytosis or endocytosis because of the small size of green-emitting QDs compared to remaining two types of QDs [51]. It has been reported that the particle size of QDs affects endocytosis or limits access to receptors of interest on cellular membranes [24,36,37]. We further examined the size effects of HER2Ab-QDs (with green, orange, and red emission) on the fluorescence imaging of HER2 receptors in KPL-4 cells.

**Figure 10.**
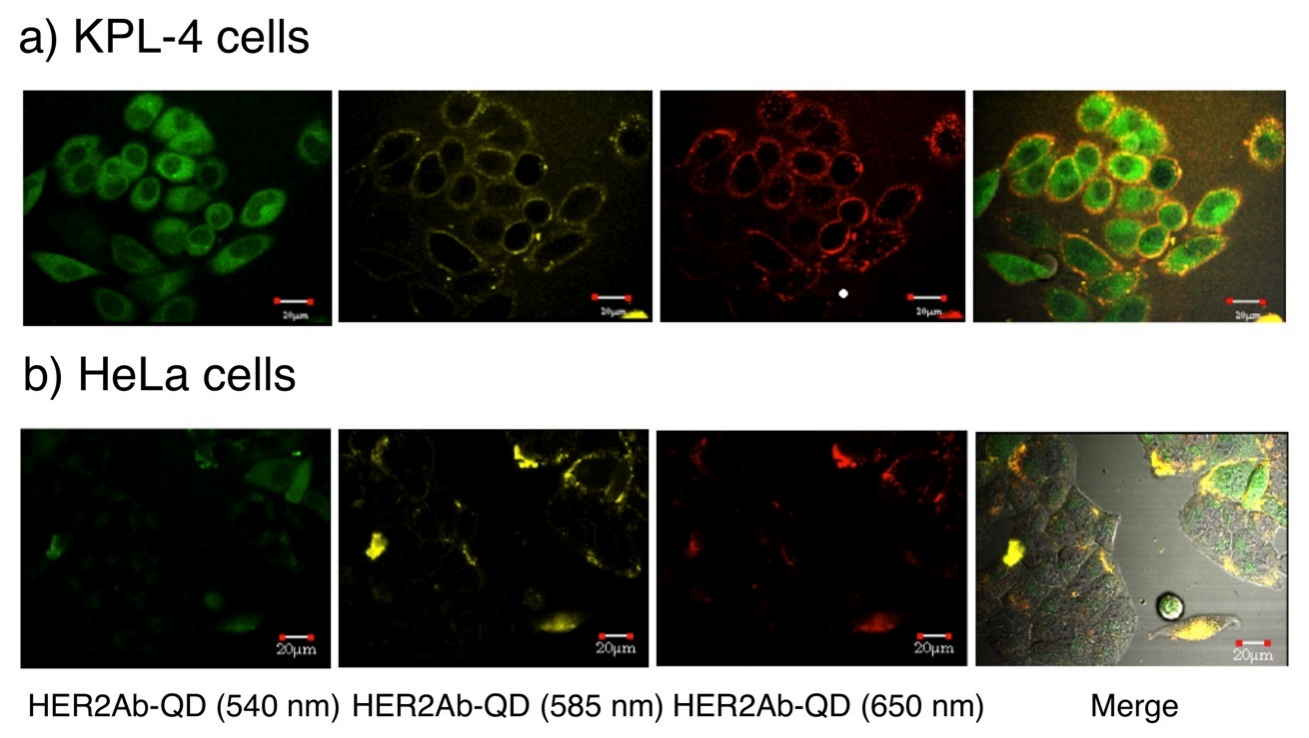
Multicolor confocal images of KPL-4 cells after incubation of HER2Ab-QDs (540 nm, 585 nm and 650 nm) prepared by SMCC coupling with reduced anti-HER2 antibodies. PBS solutions of the HER2Ab-QDs (10 nM) were incubated with KLP-4 cells for 30 min at 37 °C. QDs were excited at 405 nm. Confocal images are taken with a 490-540 nm filter for HER2Ab-QD (540 nm), a 575-620 nm filter for HER2Ab-QD (585 nm), and a 655-755 nm filter for HER2Ab-QD (650 nm). White scale bars represent 20 μm length.

**Figure 11.**
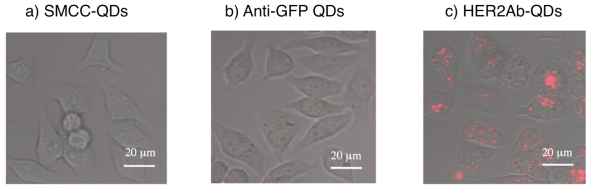
Fluorescence images of KPL-4 cells after incubation of red-emitting QDs for 1.5 hrs, with their differential interference contrast images: a) SMCC-QDs, b) anti-GFP QDs and c) HER2Ab-QDs (650 nm) prepared by SMCC coupling with reduced anti-HER2 antibodies.

Figure 10 shows the confocal images of KPL-4 and HeLa cells stained with green-, orange-, and red-emitting HER2Ab-QDs. HeLa cells are used as a negative control (expressing low levels of HER2 membrane receptors) [52]. The fluorescence images were taken after 30 min incubation of the KPL-4 and HeLa cells with HER2Ab-QDs solution (10 nM). Compared to HeLa cells, KPL-4 cells were well stained by HER2Ab-QDs due to specific binding of the QDs to HER2 receptors. Interestingly, the size of HER2Ab-QDs significantly affects on the internalization of the QDs in the KPL-4 cells (Figure 10 a). In the green-emitting HER2Ab-QDs (7 nm in diameter), the QDs are distributed at both cell membrane and cytosol. In contrast, orange- and red-emitting HER2Ab-QDs (*ca.* 12 nm each) are localized at the cell membranes. However, internalization of the orange- and red-emitting HER2Ab-QDs into cytosol was observed by further incubation of cells (>1 hour) with the QDs solution. The difference of the HER2Ab-QD staining between KPL-4 and HeLa cells indicates that the cellular uptake of HER2Ab-QDs in KPL-4 cells is receptor mediated and directed by the complex of the QDs and HER2 receptors at cell surface. The cellular uptake of red-emitting HER2Ab-QDs after 1.5 hrs incubation of KPL-4 cells is shown in Figure 11 with the control data using SMCC conjugated QDs (SMCC-QDS) and anti-green fluorescent protein antibody conjugated QDs (GFP-QDS). SMCC-QDs and anti-GFP QDs are expected to have non-binding abilities to the surface of KPL-4 cells. As can be seen from the Figure 11, the cellular uptake of QDs was specific to the HER2Ab-QDs incubated cells. SMCC-QDs and anti-GFP QDs did not stain the cell surface. This result shows that the red-emitting QDs enter to the KPL-4 cells by mediation of HER2 receptors at the cell surface. The size of HER2Ab-QDs may affect the accessibility or binding ability of the QDs to HER2 receptors present on the cell surface. The smaller HER2Ab-QDs may be rapidly taken up into the cells by their stronger binding ability to the receptors. The use of larger (red-emitting) HER2Ab-QDs is more effective for the quantitative elucidation of the HER2 expression at cell surface.

## 3. Experimental Section

### 3.1. Chemicals

Cadmium 2,4-pentanedionate (98%) was purchased from Alfa Aesar. Stearic acid, potassium *t*-butoxide, 2-mercaptoethanol and ZnEt_2_ (1M hexane solution) were purchased from Wako Chemicals (Japan). Tri-*n*-octylphosphine (TOP), tri-butylphosphine (TBP), tri-*n*-octylphosphine oxide (TOPO), hexamethyldisilathiane, and hexadecylamine (HDA) were purchased from Tokyo Kasei (Japan). Selenium (powder, 99.999%), 2-iminothiolane hydrochloride, and DL-dithiothreitol (DTT) were purchased from Sigma-Aldrich. Dimethylcadmium (10 wt% in hexane) was purchased from Strem Chemicals. 1-Ethyl-3-(3-dimethylaminopropyl)carbodiimide hydrochloride (EDC) and sulfo-succinimidyl 4-(*N*-maleimidomethyl)cyclohexane-1-carbxylate (sulfo-SMCC) were purchased from Pierce. Sulfo-NHS (3-sulfo-*N*-hydroxysuccinimide sodium salt) was purchased from Molecular Bioscience. Anti-HER2 antibody (Herceptin) was purchased from Chugai Seiyaku (Japan). Anti-GFP polyclonal antibody was kindly gifted from Medical & Biological Laboratories (Japan). Thyroglobulin (from bovine thyroid), bovine serum albumin, and ferritin (type I from horse spleen) were purchased from Sigma-Aldrich. Transferrin (apo from human) was purchased from Wako Chemicals (Japan). Other chemicals used were of analytical reagent grades.

### 3.2. QD Synthesis

#### 3.2.1. Preparation of Se (TBP) and Cd-Zn-S stock solution

All procedures were performed under argon atmosphere. One hundred mg of selenium (powder) was added to 1 mL of TBP at room temperature. Selenium was easily dissolved to TBP by sonication using a bath-type sonicator (D150H, Delta). Two mL (2 mmol) of ZnEt_2_ (1M hexane solution) and 0.57 mL (0.5 mmol) of CdMe_2_ were added to 7.0 mL of TOP. Then, 0.52 mL (2.5 mmol) of hexamethyldisilathiane was added to the CdMe_2_-ZnEt_2_/TOP solution. The stock solutions were stored in argon atmosphere.

#### 3.2.2. Synthesis of CdSe/CdZnS with a 540 nm emission peak

A mixture of 1 g of TOPO, 3 g of HDA, 66 mg of cadmium 2,4-pentanedionate, and 250 mg of stearic acid was loaded into a 25 mL three-necked flask and heated at 200 °C under an argon atmosphere. After stirring for 10 min at 200 °C, 1 mL of the stock solution of Se (100 mg/mL TBP) was swiftly added, and the temperature of the solution was lowered to 150 °C. At this temperature, the growth of CdSe QDs was monitored by measurements of their fluorescence spectra. When the emission maximum reached to 515 nm, the three-necked flask was removed from the heater and the temperature of solution was lowered to 50 °C. Then *ca.* 10 mL of ethanol was added to precipitate CdSe QDs. The precipitation of QDs was separated by centrifuge and was redissolved in 10 mL of chloroform. The QD solution was loaded into a 25 mL three-necked flask, and the chloroform was evaporated under reduced pressure. After evaporation of chloroform, 1 g of TOPO and 3 g of HDA were added to the flask and the mixture was heated to 160 °C under an argon atmosphere. After stirring for 10 min at 160 °C, 0.25 ml of a Cd-Zn-Se stock solution was added dropwise under vigorous stirring. Then the temperature of solution was decreased to 100 °C and the solution was stirred for 5 hrs at this temperature. After the temperature was lowered to 50 °C, CdSe/CdZnS QDs were precipitated by addition of excess methanol and separated by centrifuge. The precipitated QDs were redissolved to 20 mL of tetrahydrofuran.

#### 3.2.3. Synthesis of CdSe/CdZnS with a 585 nm emission peak

The mixture of 1 g of TOPO, 3 g of HDA, 66 mg of cadmium 2,4-pentanedionate, and 250 mg of stearic acid was loaded into a 25 mL three-necked flask and heated at 250 °C under an argon atmosphere. After stirring for 10 min at 250 °C, 0.6 mL of the stock solution of Se (100 mg/mL TBP) was swiftly added, and the temperature of the solution was lowered to 200 °C. At this temperature, the growth of CdSe QDs was monitored by measurements of their fluorescence spectra. When the emission maximum reached to 570 nm, the three-necked flask was removed from the heater and the temperature of solution was lowered to 50 °C. Then *ca.* 10 mL of ethanol was added to precipitate CdSe QDs. The precipitation of QDs was separated by centrifuge and was resolved in 10 mL of chloroform. The QD solution was loaded into a 25 mL three-necked flask, and the chloroform was evaporated under reduced pressure. After evaporation of the chloroform, 1 g of TOPO and 3 g of HDA were added to the flask and the mixture was heated to 180 °C under an argon atmosphere. After stirring for 10 min at 180 °C, 0.25 mL of a Cd-Zn-Se stock solution was added dropwise under vigorous stirring. Then the temperature of solution was decreased to 100 °C and the solution was stirred for 5 hrs at this temperature. After the temperature was lowered to 50 °C, CdSe/CdZnS QDs were precipitated by addition of excess methanol and separated by centrifugation. The precipitated QDs were redissolved to 20 mL of tetrahydrofuran.

#### 3.2.4. Synthesis of CdSe/CdZnS with a 650 nm emission peak

The mixture of 1 g of TOPO, 3 g of HDA, 66 mg of cadmium 2,4-pentanedionate and 250 mg of stearic acid was loaded into a 25 mL three-necked flask and heated at 320 °C under an argon atmosphere. After stirring for 30 min at 320 °C, 0.2 mL of the stock solution of Se (100 mg/mL TBP) was swiftly added, and the temperature of the solution was lowered to 300 °C. At this temperature, the growth of the CdSe QDs was monitored by measurements of their fluorescence emission spectra. When the emission maximum reached to 635 nm, the temperature was lowered to 250 °C. Then 0.25 ml of a Cd-Zn-Se stock solution was added dropwise under vigorous stirring. By addition of the Cd-Zn-Se stock solution, a red shift of *ca.*15 nm was observed in the emission spectra. Then the temperature of the solution was lowered to 100 °C and the solution was stirred for 5 hours at this temperature. After the temperature was lowered to 50 °C, QDs were precipitated by addition of excess methanol and separated by centrifugation. The precipitated QDs were redissolved in 20 mL of tetrahydrofuran.

### 3.3. Surface Modification of CdSe/CdZnS QDs with Glutathione (GSH)

To 1 mL of the tetrahydrofuran solution of CdSe/CdZnS QDs prepared according to the above method, 1 mL of GSH (200 mg/mL) was slowly added, and the mixture was heated to 60 °C. The resulting precipitates of GSH-coated QDs were separated by centrifugation and washed with water to remove tetrahydrofuran. To the QD precipitates, 1 mL of an aqueous solution (100 mg/mL) of potassium *t*-butoxide was added, and the solution was sonicated for 5 min using a bath-type sonicator. The aqueous solution of QDs was passed through a 0.2 μm membrane filter, and excess GSH and potassium *t*-butoxide were removed by dialysis (Spectrum dialysis membrane, MWCO: 50,000) using 10 mM PBS buffer. The concentration of GSH-coated QDs was estimated by using fluorescence correlation spectroscopy (FCS). The particle number of the QDs in confocal volume was measured and the concentration was estimated using an aqueous solution of rhodamine 6G (20 nM) as a reference.

### 3.4. Synthesis of Sulfo-SMCC Conjugated QDs (SMCC-QDs)

One hundred μL of sulfo-SMCC (1 mM) in water was added to 1 mL of GSH-QDs (1 μM) in PBS buffer, and the solution was incubated for 30 min. Unreacted maleimide groups of sulfo-SMCC were quenched by 5 μL of mercaptoethanol (1 mM aqueous solution). To remove unbound SMCC and mercaotoethanol, the solution was passed through a Nap™-5 column with PBS buffer as an eluent. The QD solution was centrifuged at 15,000 G for 5 min to remove aggregated QDs.

### 3.5. Conjugation of Anti-HER2 Antibody to QDs (HER2Ab-QDs)

#### 3.5.1. EDC/sulfo-NHS coupling

One hundred μL of aqueous solution (1mM) of EDC was added to 1 μL of GSH-QDs (1 μM) in 10 mM PBS buffer. Then, 250 μL of sulfo-NHS (1mM) was added to the QD solution. After 1 hour, the solution was passed through a Nap™-5 column (Sephadex G-25, GE Healthcare) with water as an eluent to remove unreacted EDC and sulfo-NHS. To the eluted solution, 1 mL of an aqueous solution of anti-HER2 antibody (1 mg/mL) was added under gentle stirring, and the solution was incubated for 1 hour for antibody conjugation at RT. Unreacted NHS groups were quenched by addition of 10 μL of mercaptoethanol (10 mM aqueous solution). Unconjugated antibodies and excess mercaptoethanol were removed by dialysis (300 kDa cellulose acetate membrane, Harvard Apparatus) using 10 mM PBS buffer. The dialyzed QD solution was passed through a 0.2 μm membrane filter to remove aggregated QDs.

#### 3.5.2. Iminothiolane/sulfo-SMCC coupling

Antibody was modified with iminothiolane for use of SMCC coupling reaction. Twenty μL of 2-iminothiolane hydrochloride (3.6 mM) was added to 1 mL of anti-HER2 antibody solution (1 mg/mL in PBS), and the mixture was incubated for 1 hr at RT. To remove unconjugated iminothiolane, the solution was passed through a Nap™-5 column with water as an eluent: this iminothiolane modified antibody solution was used for the conjugation of SMCC coupled QDs. Next, GSH-QDs was modified with sulfo-SMCC. 100 μL of sulfo-SMCC (1 mM) in water was added to 1 mL of GSH-QDs (1 μM) in PBS buffer, and the solution was incubated for 30 min. To remove unreacted SMCC, the solution was passed through a Nap™-5 column with water as an eluent. To this solution, the iminothiolane modified antibody solution was added under gentle stirring, and the mixture was incubated for 1 hr at room temperature. Unreacted maleimide groups of sulfo-SMCC were quenched by 10 μL of mercaptoethanol (10 mM aqueous solution). Unconjugated antibody and mercaptoethanol were removed by dialysis (300 kDa cellulose acetate membrane, Harvard Apparatus) using 10 mM PBS buffer. The dialyzed QD solution was passed through a 0.2 μm membrane filter to remove aggregated QDs.

#### 3.5.3. Sulfo-SMCC coupling

One mg of anti-HER2 antibody was dissolved to 1 mL of water and then 20 μL of DTT (1M) was added to the antibody solution. After 1 hr, the solution was dialyzed (Spectrum dialysis membrane, MWCO: 50,000) by using 10 mM PBS buffer. Next, GSH-QDs were modified with sulfo-SMCC. 100 μL of aqueous solution of sulfo-SMCC (1mM) aqueous solution was added to 1 mL of GSH-QDs (1 μM, PBS buffer), and the mixture was incubated for 30 min at RT. To remove unreacted SMCC, the solution was passed through a Nap™-5 column with water as an eluent. To this solution, 1 mL of the reduced antibody (*ca.* 1 mg/mL) was added under gentle stirring, and the solution was incubated for 1 hour for antibody conjugation. Unreacted maleimide groups of sulfo-SMCC were quenched by 10 μL of mercaptoethanol (10 mM aqueous solution). Unconjugated antibodies and excess mercaptoethanol were removed by dialysis (300 kDa cellulose acetate membrane, Harvard Apparatus) using 10 mM PBS buffer. The dialyzed QD solution was passed through a 0.2 μm membrane filter to remove aggregated QDs.

### 3.6. Synthesis of Anti-GFP Polyclonal Antibody Conjugated QDs (anti-GFP QDs)

Fifty μg of anti-GFP polyclonal antibody was dissolved to 100 μL of water and then 1 μL of DTT (1M) was added to the antibody solution. After 1 hr, the solution was dialyzed (Spectrum dialysis membrane, MWCO: 50,000) using 10 mM PBS buffer. Next, GSH-QDs were modified with sulfo-SMCC. Five μL of aqueous solution of sulfo-SMCC (1 mM) aqueous solution was added to 50 μL of GSH-QDs (1 μM, PBS buffer), and the mixture was incubated for 30 min at RT. To remove unreacted SMCC, the solution was passed through a Nap™-5 column with water as an eluent. To this solution, 100 μL of the reduced antibody (*ca.* 0.5 mg/mL) was added under gentle stirring, and the solution was incubated for 1 hr for antibody conjugation. Unreacted maleimide groups of sulfo-SMCC were quenched by 5 μL of mercaptoethanol (1 mM aqueous solution). Unconjugated antibodies and excess mercaptoethanol were removed by dialysis (300 kDa cellulose acetate membrane, Harvard Apparatus) using 10 mM PBS buffer. The dialyzed QD solution was passed through a 0.2 μm membrane filter to remove aggregated QDs.

### 3.7. Characterization of QDs

Fluorescence spectra of QDs were measured with a FP-6200 spectrofluorometer (JASCO). Emission efficiencies of QDs were evaluated as absolute quantum yields using a quantum yield measurement system (C10027, Hamamatsu Photonic). The absolute quantum yield (*QY*) is defined as *QY* = *PN_em_/PN_ab_*, where *PN_em_* and *PN_ab_* are the number of emitted and absorbed photons by fluorescence materials. Excitation wavelengths were set to 440 nm for green-emitting QDs (540 nm emission), and 488 nm for orange-emitting and red-emitting QDs (540 nm and 650 nm emission).

The hydrodynamic diameters of QDs were evaluated by using dynamic light scattering (DLS) and fluorescence correlation spectroscopy (FCS). DLS data were collected by using a Malvern DLS apparatus (Nano-ZS) with a 633 nm He/Ne laser. Fluorescence autocorrelation curves were measured by using a compact FCS system (C9413-01MOD, Hamamatsu Photonics) with a 473 nm semiconductor laser as an excitation light source.

FCS uses the fluctuations of the fluorescence intensity in a tiny confocal volume to determine the diffusion times of fluorescent particles [52]. Fluctuations in the fluorescence intensities *I(t)* can be analyzed by using the autocorrelation function *G̣τ)*:

(1) $$G(\tau )=\frac{<I(t)I(\tau +t)>}{{<I(t)>}^{2}}$$

where the symbol < > stands for the ensemble average.

If a three-dimensional Gaussian profile in the confocal volume of the lateral radius *ω_o_* and axial radius *ω_z_* was assumed, equation (1) for a one-component diffusion can be expressed as:

(2) $$G(\tau )=1+\frac{1}{N}{\left(1+\frac{\tau }{\tau_{d}}\right)}^{-1}{\left(1+\frac{\tau }{{\left(\omega_{z}/2\omega_{o}\right)}^{2}\tau_{d}}\right)}^{-1/2}$$

where, *τ_d_* = *ω_o_^2^/4D*. *N* is the average number of fluorescent particles in the excitation volume and *τ_d_* is the diffusion time of the fluorescent particles, depending on the diffusion constant *D* and *ω_o_*. Using the Stokes-Einstein relationship (*D* = *k_B_T/6pηr*), the hydrodynamic diameter *d_QD_* of QDs can be calculated from the following equation:

(3) $$d_{QD}=d_{ST}\times \frac{\tau_{QD}}{\tau_{ST}}$$

where *d_ST_* is the diameter of a standard particle, and *τ_QD_* and *τ_ST_* are the diffusion times of QDs and the standard particles, respectively. As a standard particle, we used fluorescent latex beads (20 nm in diameter, Molecular Probe, Inc.).

The images of atomic force microscopy (AFM) were acquired with SPI-3800N (Seiko Instruments Inc.) and Si cantilever (f=134 kHz, C=16 N/m, Seiko Instruments Inc.) in tapping mode. A drop of QD solution (10 nM) was spotted onto fleshly cleaved mica (Nilaco Corporation) and was blown off after 1min. Then the QD sample was subjected to air-drying for 12 hrs.

Size exclusion column chromatography was performed with a HPLC system (Hitachi L-2130 and L-2400) using TSKgel G4000SW column (7.8 mm×30 cm, TOSOH, Japan). Twenty mL of QD solution (:10 mM PBS) was injected to the column and eluted by 10 mM PBS at room temperature with a flow rate of 1 mL/min.

### 3.8. Cell Lines

KPL-4 human breast cancer cell line overexpressing HER2 and HeLa cell line were subjected to fluorescence imaging. The KPL-4 cell line was kindly provided by Prof. J. Kurebayashi (Kawasaki Medical School, Kurashiki, Japan) and Prof. N. Ohuchi (Tohoku University, Japan) [54,55]. HeLa cell line was used as a negative control of HER2. Both cell lines were grown in four chamber glass bottom plates (LabTek, USA) containing Dulbecco's modified Eagle's medium (DMEM, Sigma-Aldrich, St. Louis, MO, USA) with 10% fetal bovine serum, 100 mg/mL penicillin, and 10 mg/mL streptomycin. Culture plates of both cell lines (∼75% growth) were used for labeling with HER2Ab-QDs. Old culture medium of all plates were replaced with 300 μL of HER2Ab-QDs (10 nM) containing DMEM culture medium (phenol red and FBS free). All treated plates were incubated for 30 min at 37 °C and the cells were washed two times with PBS buffer. Three hundred μL phenol red free DMEM media were filled in all plates and cells were examined under confocal laser fluorescence microscopy. For other types of QDs for cell staining, similar procedures with the case of HER2Ab-QDs were performed.

### 3.9. Confocal Fluorescence Microscopy

Confocal fluorescence images were taken with an Olympus confocal inverted microscope (FluoView1000) using an oil immersion objective lens (60 ×, N. A. = 1.35). Excitation was performed at 405 nm with a LD pumped solid laser. Emission filters of 490-540 nm, 575-620 nm, and 655-755 nm were used for the cells incubated with green-, orange-, and red-emitting HER2Ab-QDs.

## 4. Conclusions

We have synthesized three types of HER2Ab-QDs based on EDC/sulfo-NHS, iminothiolane/sulfo-SMCC, and sulfo-SMCC coupling reactions for fluorescence imaging of HER2 overexpressing cancer cells. To conjugate antibody to QDs, we used GSH coated QDs with high quantum yields (0.23−0.39). Hydrodynamic size of the HER2 conjugated QDs depended on the coupling reaction for antibody conjugation, and the values of the size were ranging from 9.4 nm to 12 nm in diameter. AFM images of the HER2Ab-QDs indicate that the number of antibody molecules conjugating to the QD surface is limited by the steric hindrance between antibody molecules.

Fluorescence images of HER2 expression in KPL-4 breast caner cells showed that the colloidal stability of HER2Ab-QDs prepared by SMCC coupling are higher than that of HER2Ab-QDs prepared by remaining two coupling methods. In addition, the size of HER2Ab-QDs significantly affects on the fluorescence imaging of HER2 expression in the KPL-4 cells. Smaller (green-emitting) HER2Ab-QDs were rapidly taken up into the inside of the cells by endocytosis or macropinocytosis, while larger (red-emitting) HER2Ab-QDs were distributed to the cell surface and slowly taken up. The red-emitting (650 nm) HER2Ab-QDs (prepared by using SMCC coupling to reduced antibodies) are most effective and quantitative for imaging of HER2 expression in the breast cancer cells.

The fluorescence imaging of HER2 expression can be performed by using a very low concentration (10 nM) of HER2Ab-QDs. Compared with conventional IHC and FISH, the QD-based HER2 detection method is easy, fast and economic. Since QDs are resistant to photobleaching, quantitative evaluation of HER2 expression may be possible by using HER2Ab-QDs. The use of HER2Ab-QDs as a fluorescent probe has great potential for the precise determination of HER2 status of breast cancer patients in cellular levels.

## Figures and Tables

**Table 1.** Fluorescence quantum yields, hydrodynamic diameters, and apparent molecular weights of GSH-QDs in 10 mM PBS buffer.

	**GSH-QD (540 nm)**	**GSH-QD (585 nm)**	**GSH-QD (650 nm)**
Quantum yield	0.33 *^a^*	0.23 *^b^*	0.39 *^b^*
Diameter (nm)	4.5 ± 0.6 *^c^*	5.9 ± 0.5 *^c^*	Not determined
	4.4 ± 0.2*^d^*	5.1± 0.3 *^d^*	6.9 ± 0.5 *^d^*
Apparent MW (kDa)	75	150	300

a Excitation: 440 nm,

b excitation: 488 nm,

c evaluated by DLS,

d evaluated by FCS
